# Mapping of five candidate sex-determining loci in rainbow trout (*Oncorhynchus mykiss*)

**DOI:** 10.1186/1471-2156-10-2

**Published:** 2009-01-15

**Authors:** Mahmoud A Alfaqih, Joseph P Brunelli, Robert E Drew, Gary H Thorgaard

**Affiliations:** 1 School of Molecular Biosciences, Washington State University, Pullman WA 99164-4234, USA; 2 Department of Pharmacology and Physiology, Mutah University, Karak 61710, Jordan; 3 School of Biological Sciences and Center for Reproductive Biology, Washington State University, Pullman WA 99164-4236, USA; 4 University of Idaho, Department of Biological Sciences, Moscow, ID 83844-3051, USA

## Abstract

**Background:**

Rainbow trout have an XX/XY genetic mechanism of sex determination where males are the heterogametic sex. The homology of the sex-determining gene (SDG) in medaka to *Dmrt1 *suggested that SDGs evolve from downstream genes by gene duplication. Orthologous sequences of the major genes of the mammalian sex determination pathway have been reported in the rainbow trout but the map position for the majority of these genes has not been assigned.

**Results:**

Five loci of four candidate genes (*Amh*, *Dax1*, *Dmrt1 *and *Sox6*) were tested for linkage to the Y chromosome of rainbow trout. We exclude the role of all these loci as candidates for the primary SDG in this species. *Sox6i *and *Sox6ii*, duplicated copies of *Sox6*, mapped to homeologous linkage groups 10 and 18 respectively. Genotyping fishes of the OSU × Arlee mapping family for *Sox6i *and *Sox6ii *alleles indicated that *Sox6i *locus might be deleted in the Arlee lineage.

**Conclusion:**

Additional candidate genes should be tested for their linkage to the Y chromosome. Mapping data of duplicated *Sox6 *loci supports previously suggested homeology between linkage groups 10 and 18. Enrichment of the rainbow trout genomic map with known gene markers allows map comparisons with other salmonids. Mapping of candidate sex-determining loci is important for analyses of potential autosomal modifiers of sex-determination in rainbow trout.

## Background

Teleost fishes have a variety of evolutionarily labile sex-determination systems and relatively undifferentiated sex chromosomes [1], and thus are interesting research models for the exploration of how sex-determining mechanisms evolve. The current view of the sex-determination cascade describes it as a gene hierarchy [2]. Genes that reside on top of this hierarchy have been recruited only relatively recently [3] and are only found in specific branches of the phylogenetic tree. Downstream genes are conserved between the different phyla, regardless of the sex-determination mechanism, and thus seem to have been present for a much longer time. The recent discovery that the sex-determining gene of the medaka, *Dmy*, is a homologue of a downstream gene of the pathway in mammalian species [4] led researchers to propose that downstream genes can be recruited to the top of the hierarchy by gene duplication [5]. This model of evolution of the sex-determining gene cascade is further supported in the tilapia model (*Oreochromis *spp) where the two downstream genes in mammals, *Amh *and *Dmrta2*, map within quantitative trait loci, QTL, regions for sex determination in that genus [6].

The salmonid fish, rainbow trout (*Oncorhynchus mykiss*) has several parallels to the medaka. Both have an XX-XY genetic sex-determination system with the male being the heterogametic sex [7-9] and in both species the sex chromosomes are at an early stage of evolution [8-12]. The sex-determining mechanism in the rainbow trout is believed to function via a 'dominant' factor located on the Y chromosome as evidenced by the production of XXY triploid males [13]. *Dmy*, the sex-determining gene in the medaka, also appears to function as a dominant factor. This is supported by male phenotypic development of genetically female (XX) medaka fish injected with a genomic clone containing the *Dmy *sequence [14].

Based on the proposed model for evolution of sex-determining gene cascades, we hypothesized that in a tetraploid-derived organism like the rainbow trout [15] which has a high repertoire of duplicated genes, any gene in the sex differentiation cascade, or its' homologue, could potentially have evolved as a sex- determining gene. Based on what we currently know from the two sex-determining genes discovered to date in vertebrates, *Sry *in mammals and *Dmy*, in the medaka, a sex-determining gene (1) has an upstream position in the cascade, (2) shows stronger expression in male gonads as compared to female, (3) is expressed before sexual differentiation of the gonad and (4) is linked to the Y chromosome. We investigated several candidate genes for sex determination in the rainbow trout model by testing for Y-linkage.

Orthologous sequences of the major genes of the mammalian sex determination pathway have been reported in the rainbow trout; including *Wt1*, *Sox9*, *Dmrt1*, *Amh *and *Dax1*. Three *Wt1 *loci [16], and three *Sox9 *loci (Alfaqih et al. Submitted) have been previously mapped by our laboratory, but none of the loci were linked to the Y chromosome. In the present study, we tested for linkage of four additional candidate genes for sex determination (*Sox6*, *Dmrt1, Dax1 and Amh*) to the Y chromosome of rainbow trout. As we will show, our results have eliminated these loci as candidates for the primary sex-determining gene in the rainbow trout. We also identified a duplication of *Sox6 *and discuss the inheritance of the duplicated *Sox6 *loci in our mapping families. In addition, we report the complete genomic sequence of *Dax1 *and a promoter subsequence analysis of *Dax1 *and *Dmrt1 *that implicates the role of *Pax2 *in trout testicular differentiation.

## Results and discussion

### Linkage analysis of *Amh*, *Dax1 *and *Dmrt1*

**Figure 1:**
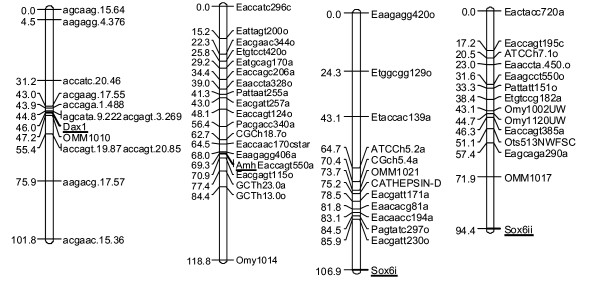
**Genetic map of rainbow trout (*Oncorhynchus mykiss*) candidate sex-determining loci**. Numbers on the left indicate distances in centimorgans. Marker names are indicated on the right. The linkage group number and the mapping family used for linkage analysis are indicated on top of each linkage group. Candidate sex-determining loci are underlined.

Through comparison of rainbow trout *Amh *cDNA sequence with zebrafish (*Danio rerio*) orthologous genomic sequences, it was determined that the *Amh *gene of rainbow trout and zebrafish both have six tentative introns. We amplified, cloned and sequenced a 1.35 kb genomic fragment that contained the 3^rd^, 4^th^, 5^th ^and 6^th ^introns of *Amh *from the OSU and Arlee rainbow trout clonal lines (the presence of the 1^st ^and 2^nd ^introns remains tentative and still requires experimental verification). Intron sizes were 337 bp, 87 bp, 105 bp and 91 bp respectively. Comparison of OSU and Arlee intron sequences indicated the presence of a SNP in the 5^th ^intron of OSU in a *Tsp509I *restriction site (Table 1). PCR-RFLP with *Tsp509I *was used to genotype doubled haploids of the O × A mapping family for receiving either allele. Linkage analysis assigned *Amh *to linkage group 8 (Figure 1), which corresponds to the short arm of chromosome 5 in the karyotype [17].

**Table 1:** Typing of *Amh, Dax1 *and *Dmrt1 *genes and alleles by sequence polymorphisms in rainbow trout crosses.

Gene	Accession numbers	Parent	Site of polymorphism	Polymorphism	Method of genotyping
*Amh*	FJ609190	Arlee	82–85 of fifth intron	AAT(T)	PCR-RFLP *Tsp509I*
	FJ609189	OSU	82–85 of fourth intron	AAT(G)	
*Dax1*	FJ617280	HC	-10 of 5' UTR	**G**	Taqman assay
	FJ617279	OSU	-10 of 5' UTR	**A**	
*Dmrt1*	--	HC	743 of first intron	GTA**(C)**	PCR-RFLP *Rsa1*
	FJ617281	OSU	743 of first intron	GTA**(A)**	

Prior to this study, the sequence of the first exon and part of the sequence of the second exon of rainbow trout *Dax1 *was deposited in nucleotide databases as an expressed sequence tag, EST [Genbank: BX298095]. In order to look for sequence polymorphisms, more likely to be found in non-coding regions of the gene, we isolated and sequenced a complete genomic clone of the *Dax1 *gene [Genbank: FJ617278]. Tentative locations of the start codon, stop codon and exon-intron boundaries were inferred based on sequence alignment with cDNA sequences of *Dax1 *from *Dicentrarchus labrax *[Genbank: AJ633646] and *Oreochromis niloticus *[Genbank: AY135397] and were not experimentally verified. Our sequence alignment indicated that the coding region of *Dax1 *contains an ORF of 909 bp and is organized into two exons (670 and 239 bp in size respectively) separated by a 560 bp intron. A 303 amino acid polypeptide was deduced from the inferred cDNA sequence.

No polymorphism between parental lines of our mapping family was detected in the intron, thus a 1.1 kb fragment of the genomic region located upstream to the putative coding sequence of *Dax1 *was amplified and sequenced to look for sequence polymorphisms. An (A/G) SNP between OSU and HC parental lines was found in the sequenced region (Table 1). Doubled haploids of the O × H mapping family were genotyped for either allele. Genotyping doubled haploids mapped *Dax1 *to linkage group 5 (Figure 1), which corresponds to the long arm of chromosome 22 on the physical map [17].

Sequence analysis revealed the presence of a SNP between OSU and HC that resulted in the loss of an *RsaI *site in the HC allele in a 1.89 Kb genomic fragment containing the first intron of *Dmrt1 *(Table 1). PCR-RFLP using *Rsa1 *restriction was used to genotype doubled haploids of the O × H mapping family for receiving either *Dmrt1 *allele but linkage analysis was unable to assign *Dmrt1 *to any linkage group in the O × H map. Chi-square analysis did not support linkage of the HC (YY) *Dmrt1 *allele with the Y chromosome of rainbow trout as indicated by phenotypic sex of the doubled haploid fish (χ2 = 5.241, d.f. = 3, α = 0.05). One explanation for the failure to map *Dmrt1 *is the absence of markers closely linked with *Dmrt1 *in the genetic map. A second explanation would be that a duplicate of this gene may have also been amplified and interfered with accurate genotyping. However, a chi-square test (χ2 = 2.572, d.f. = 1, α= 0.05) did not detect deviation from Mendelian ratios which would likely occur in such a situation.

### Promoter Subsequence Analysis of *Dax1 *and *Dmrt1*

Subsequence analysis of an upstream 1.2 Kb promoter sequence of *Dax1 *[Genbank: FJ617278] and a 649 bp promoter sequence of *Dmrt1 *[Genbank: FJ617281] identified a number of transcription factor binding sites or *cis *regulatory elements (Table 2 and Table 3). Some of these elements are binding sites for products of genes known to be involved in the sex differentiation cascade of mammals including elements for SF1 (steroidegenic factor 1), WT1 (Wilms' tumor suppressor), COUP-TF (chicken ovalbumin upstream promoter-transcription factor), HMGA (high mobility group A) and PAX (paired domain box). Experiments in mammalian systems have demonstrated the interaction of the *Dax1 *gene product with SF1, WT1, and COUP-TF [18-20].

**Table 2:** Promoter sequence analysis of rainbow trout *Dax1*.

Element	Sequence	Location (bp)	Strand
PAX5	CTGTAGCACTGAAATGCAGTGCCTTAGAC	-1096/-1124	+
SF1	ATCTCAAGGCCAT	-816/-828	+
COUP-TF	CCGAGTGGGGCAGCGGTCTAAGGCA	-1082/-1105	-
HMGA	ACAAATTCACAACTCCC	-523/-539	-
TATA box	TAATATAAATGTGCTGC	-123/-139	+
WT1	TTGCGCGGGGGTATCAG	-104/-119	+
PAX 2/5/8	CTTTGAAGAGTTG	-52/-63	-
SOX-5	TTCTGACAATGCAGTAA	-488/-504	-

Nucleotide positions correspond to the start codon.

**Table 3:** Promoter sequence analysis of rainbow trout *Dmrt1*.

Element	Sequence	Location (bp)	Strand
SF1	CCTACAAGGTTAC	-176/-188	-
SF1	TTTGCAAGGCCAC	-438/-450	+
PAX2	CAACTTCTCGCGACATTAAACAC	-138/-160	+
PAX3	ACTGTCCCACGTGTACTCT	-187/-205	-
PAX6	ATTAAACACTTCAATAAGA	-128/-146	+

Nucleotide positions correspond to the start codon.

### Duplication and linkage analysis of *Sox6*

Boundaries of eight putative introns were identified in *Sox6 *of rainbow trout by comparing the cDNA sequence from rainbow trout to genomic sequences of zebrafish (*Danio rerio*) and fugu (*Takifugu rubripes*). PCR amplification of the fourth intron of *Sox6 *from OSU and HC produced two products of differing size in each of the clonal lines. Following cloning and sequencing of all four products, OSU products were determined to be 984 and 1067 bp in size, while the sizes of the recovered products in HC were 984 and 1097 bp (Table 4). Using the BLASTN algorithm, all four sequences were verified to be that of *Sox6*. The larger-sized OSU and HC alleles were named *Sox6i *while the smaller-sized alleles were named *Sox6ii*. Sequence analysis of the four different *Sox6 *alleles in OSU and HC revealed the presence of a microsatellite repeat nested in the 4^th ^intron. The size difference between *Sox6i *and *Sox6ii *is due to an expansion in the size of the repeat (Table 4). The size difference between the HC and OSU alleles at *Sox6i *is due to a 30 bp expansion of the microsatellite repeat in HC. No size or sequence polymorphisms were detected between OSU and HC at *Sox6ii*. The presence of two *Sox6 *products in each of the homozygous clonal lines (OSU and HC) indicated that *Sox6 *is duplicated in the rainbow trout.

**Table 4:** Typing of *Sox6 *genes and alleles by sequence polymorphisms in rainbow trout crosses.

Gene	Accession numbers	parent	Site of polymorphism	Polymorphism	Method of genotyping	Size of marker
*Sox6ii*	EU848567	Arlee	725–729 of fourth intron	G**(G)**ACC	PCR-RFLP *Ava II*	984 bp
*Sox6i*	EU848564	OSU	806–810 of fourth intron	G**(G)**ACC		1067 bp
*Sox6ii*	EU848565	OSU	725 of fourth intron	G**(A)**ACC		984 bp
*Sox6i*	EU848568	HC	Size of fourth intron	30 bp expansion	Size polymorphism	1097 bp
*Sox6ii*	EU848569	HC	No polymorphism with *Sox6ii *of OSU			984 bp

**Figure 2:**
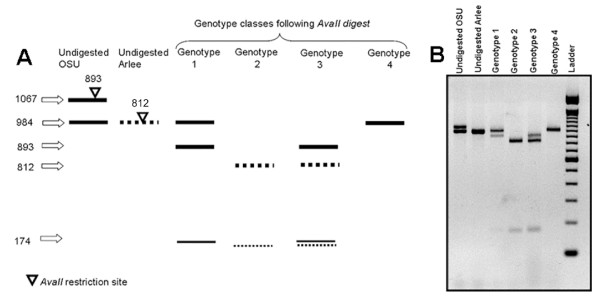
**Inheritance of *Sox6 *alleles in the rainbow trout OSU × Arlee mapping family**. (A) A schematic diagram showing the different *Sox6 *alleles in OSU and Arlee parental lines and the different genotype classes that were observed in doubled haploids of the OSU × Arlee mapping family. The OSU alleles are shown in solid lines while the Arlee alleles are shown in dashed lines. Restriction sites of *AvaII *enzyme are indicated by an inverted triangle on each respective *Sox6 *allele. The distance of each restriction site away from the 5' end of each respective allele is indicated above the triangle. Sizes of the different *Sox6 *alleles before their digestion with *AvaII *and the sizes observed in the mapping family following *AvaII *digestion are indicated with arrows. (B) A 2% agarose gel image of the different *Sox6 *alleles in OSU and Arlee parental lines and the different genotype classes observed in doubled haploids created by androgenesis from an OSU × Arlee hybrid. Look at part (A) for details.

PCR amplification of the same intron from Arlee produced only one product 984 bp in size. Cloning and sequencing of the product did not show any sequence variants supporting the presence of a second *Sox6 *allele in Arlee. Sequence comparison revealed a polymorphism between OSU and Arlee parental lines that resulted in the loss of an *AvaII *restriction site in *Sox6ii *of OSU (Table 4 and Figure 2(A)). PCR-RFLP with *AvaII *was used to genotype doubled haploids of the O × A mapping family (refer to Materials and Methods under linkage mapping for details on genotyping doubled haploids).

In the O × A mapping family, linkage analysis assigned *Sox6i *to linkage group 18 (acrocentric chromosome 26) and *Sox6ii *to linkage group 10 (long arm of chromosome 6) supporting their homeology (Figure 1). This relationship was previously suggested by linkage analysis of duplicated microsatellites and by physical mapping of bacterial artificial chromosome (BAC) clones of duplicated loci [17,21,22]. Since there were no polymorphisms in the recovered *Sox6ii *sequences between OSU and HC, this allele could not be mapped in the O × H cross. The size polymorphism in *Sox6i *between OSU and HC allowed us to map this gene to linkage group 18, in agreement with results from the O × A cross.

### Inheritance of *Sox6 *alleles in the O × A family

Two models are considered here to explain the inheritance pattern of *Sox6 *loci in individuals of the O × A mapping family.

#### 1. Simple disomic inheritance with identical *Sox6 *Arlee alleles

If we assume that Arlee (A) has two identical *Sox6 *alleles and assume independent assortment and random segregation of OSU and Arlee alleles, four genotype classes should be observed: (1) OSU – *Sox6i*/OSU – *Sox6ii *(Genotype 1 in figure 2) (2) A – *Sox6i*/A – *Sox6ii *(Genotype 2) (3) OSU – *Sox6i*/A – *Sox6ii *(Genotype 3) (4) A – *Sox6i*/OSU – *Sox6ii*. This last genotype was not observed in the O × A family (figure 2) which raises questions regarding validity of the model.

#### 2. Simple disomic inheritance with failure to amplify *Sox6i *allele from Arlee

If we assume that primer combination used to amplify different *Sox6 *loci failed to amplify *Sox6i *allele in Arlee because of a mismatch in the primer sequence, or because of deletion of *Sox6i *in the Arlee lineage, *Sox6i *allele in Arlee can be genotyped as a null allele. Four genotype classes are expected in the O × A progeny if *Sox6i *is genotyped as a null allele: (1) OSU – *Sox6i*/OSU – *Sox6ii *(Genotype 1 in figure 2) (2) A – *null*/A – *Sox6ii *(Genotype 2) (3) OSU – *Sox6i*/A – *Sox6ii *(Genotype 3) (4) A -*null*/OSU – *Sox6ii *(Genotype 4). Genotype analysis of the O × A mapping family showed that 23 out of 73 doubled haploids were of genotype 1, 20 doubled haploids were of genotype 2, another 20 doubled haploids were of genotype 3 and only 9 were of genotype 4. Chi-square analysis did not show significant deviation of the observed proportions for the different genotype classes from the theoretical proportions expected under this model (χ2 = 6.2603, d.f. = 3, α = 0.05).

The (A -*null*/OSU – *Sox6ii*) genotype can also be explained by failure of the *AvaII *restriction assays. Genotyping was however confirmed by digestion with *NlaIII *which also produces a distinctive restriction profile for each of the four genotype classes of the mapping family and thus ruling out this possibility (data not shown).

### Additional candidate genes for sex-determination

We tested five loci for their linkage to the Y chromosome of rainbow trout in this study. In addition, three *Sox9 *loci (Alfaqih et al. Submitted) and three *WT1 *loci [16] have also been mapped in rainbow trout. This brings the total number of candidate genes of rainbow trout sex determination tested for linkage to the Y chromosome to eleven. All of the loci tested were chosen based on their role in sex determination in mammals and on expression during testicular differentiation of rainbow trout [23].

A number of candidate genes can still be tested for their linkage to the sex locus of rainbow trout. In mammalian species, ten out of the 30 genes discovered so far in the *Sox *gene (*Sry*-related HMG box) family have been shown to be involved in the sex differentiation pathway or expressed in embryonic testes or ovaries [24,25]. Rainbow trout orthologs of five of those genes have been deposited in GENBANK or the TIGR Rainbow Trout Gene Index. Genes of the DM domain (Doublesex and MAB-3 related) family are also strong candidates for sex determination in rainbow trout. There are at least six DM domain genes in fishes, most of them with unknown functions [24,26]. In addition to *Dmrt1*, orthologous sequences of *Dmrt2 *and *Dmrt4 *have been isolated from rainbow trout [27]. Studying the gene expression profile of 102 genes involved in gonadal differentiation of rainbow trout, Baron et al [23] identified *Pax2a *as a gene that is expressed early in gonadal development and displays a testis-specific expression profile. Our study identified potential binding sites of *Pax2 *in the promoter sequences of *Dmrt1 *and *Dax1 *suggesting that members of this gene family should be explored in future analyses. Although our analysis focused on candidate genes that already have sequences deposited in public databases, paralogous loci might still be isolated and would also be strong candidates for sex-determination based on the model presented for evolution of sex-determining systems [2].

It is largely accepted that the sex-determining system in rainbow trout is genetic with male heterogamety. Nonetheless, Quillet et al [28] reported an equal ratio of males and females in a mitotic gynogenetic family. Studying the transmission of maleness of XX gynogenetic fish across the next three generations, the authors concluded that unexpected maleness might be caused by a recessive mutation of a sex-influencing factor carried on an autosomal pair of chromosomes. This conclusion however was not accompanied by any genetic mapping of the causative mutation. Our mapping data of the candidate genes will thus be of great value in analysis of the inheritance of such an autosomal sex-determining factor.

## Conclusion

Using linkage analysis, we excluded the role of five candidate loci as the primary sex-determining gene in rainbow trout. We also identified a duplication of the *Sox6 *locus. Inheritance pattern of *Sox6 *loci in O × A mapping family indicated possible deletion of *Sox6i *locus in the Arlee lineage. Our mapping data supports previously suggested homeology between linkage groups 10 and 18 in rainbow trout, and adds four known genes to its genomic map. Enrichment of the rainbow trout genomic map with type I markers allows the identification of conserved syntenic blocks between rainbow trout and other salmonid species, which would eventually allow for understanding the molecular organization of genomes following genome duplication. Although we did not find the primary sex-determining gene in this study, our data is of importance for analyses of potential autosomal modifiers of sex-determination in rainbow trout.

## Methods

### Mapping families and linkage maps

Two mapping families and their respective linkage maps have been used for this study. Mapping families are doubled haploid fish generated from F1 hybrids between two clonal lines using androgenesis. Clonal lines were produced by androgenesis and gynogenesis [29,30]. Each clonal line is homozygous at all loci and its isogenecity has been confirmed by multilocus DNA fingerprinting [31].

#### OSU × HC mapping family (O × H)

This family was produced from the F1 hybrid of the Oregon State University (OSU: XX female) and Hot Creek (HC: YY male) clonal lines. The family was previously described in Zimmerman et al [32] where it was used to identify a single major QTL affecting natural killer cell-like activity. In our study, 85 doubled haploid fish from the original mapping family were genotyped for polymorphisms in non-coding regions of the candidate genes *Sox6*, *Dmrt1 *and *Dax1*.

#### OSU × Arlee mapping family (O × A)

This family was produced by androgenesis from a cross between the OSU (XX) and Arlee (AR; YY) clonal lines, previously used to generate a dense linkage map and is described in Young et al [33] and Nichols et al [22]. In our study, 76 doubled haploid fish of the original mapping family were genotyped for polymorphisms in non-coding regions of the candidate genes *Sox6 *and *Amh*. Synteny between the O × H and O × A linkage maps has been established [32].

### Identification of polymorphisms in non-coding regions of candidate genes

#### Primer design

In order to amplify genomic DNA fragments of non-coding regions of candidate genes, primers were designed to anneal within exons flanking tentative introns. Boundaries between exons and introns were predicted for *Sox6*, *Dmrt1 *and *Amh *through comparisons of their cDNA sequences from Genbank (*Dmrt1*; AF209095 and *Sox6*; D61688) or TIGR gene index (*Amh*; TC103383) with genomic sequences of *Takifugu rubripes *and *Danio rerio*. Primer sequences are listed in Table 5.

**Table 5:** Primer sequences for amplifying non-coding regions of candidate sex-determining genes in rainbow trout.

Gene	Cloning/Library screening primers	Marker position	Mapping primers
*Sox6*	F: TTCACAGGCAGCAAGACCAG R: AACAGCGCTGTGGAGTTCAG	4^th ^intron	MF: TTCACAGGCAGCAAGACCAG MR: AACAGCGCTGTGGAGTTCAG
			
			SF: CTAGAGCTGCGGATGTTGTAAC SR: TGAGCCCACTGGCAGGTGTTC
			
*Amh*	F: CATCACTTTCACCAGTCACTC R: TCGGTACTGCGTCTCACTG	4^th ^intron	MF: CATCACTTTCACCAGTCACTC MR: TCGGTACTGCGTCTCACTG
			
*D*ax1	LF: CTCCGGTCACCGCAGGTTAC LR: AGGATCCGTTGCAACATGC	5' UTR	TF: CGAGCAGCACCCGATGTAG TR: CCTCGCGAGTGGCCAT
			
	F: ACCTACAGCACCGAATATCAC R: GTTGTTGCCTTAGCTCAAGC		P: CACGTGGTGCGCG P: CACGTGGCGCGCG
			
*Dmrt1*	F: AGGAACCACGGCTACGTGT R: CAACCTCCTGACTGGACAG	1^st ^intron	MF: CAGAAATGCAAACTGATCGC MR: CAACCTCCTGACTGGACAG

F and R, forward and reverse primers used for amplifying genomic fragments that were later cloned and sequenced; LF and LR, forward and reverse primers used for library screening; MF and MR, forward and reverse primers used for mapping each respective fragment; SF and SR; forward and reverse primers used for genotyping by size polymorphisms; TF and TR: forward and reverse primers used for genotyping by Taqman assay. P: Sequences of the probes used for genotyping by Taqman assay.

### PCR reactions

All PCR reactions were performed using a Thermolyne Amplitron II thermocycler. PCR reaction conditions began with denaturation at 94°C for 2 minutes, followed by 30 cycles of 94°C for 45 seconds, 60°C for 45 seconds and 72°C for 45 seconds. The reaction ended with a final extension step of 72°C for 2 minutes. All reactions were 20 μl in volume containing: 50 ng DNA template, 1× PCR buffer (Invitrogen, Carlsbad, CA), 2.5 mM MgCl_2_, 10 pmoles of each primer, 0.25 mM dNTPs (Invitrogen, Carlsbad, CA) and 1.5 U of Taq DNA Polymerase (Promega, Madison, WI).

#### Cloning, sequencing and sequence alignments

Following the amplification of non-coding regions of candidate genes, PCR amplification products were resolved in a 2% agarose gel stained with ethidium bromide. Desired DNA fragments were gel-purified using Gene Clean (Bio 101, Vista, California), cloned into the pGEM-T Easy plasmid system (Promega, Madison, WI) and then sequenced at the Washington State University Laboratory for Biotechnology and Bioanalysis. Target gene sequence verification was performed using the BLASTN and BLASTX algorithms of the NCBI website. Sequence polymorphisms between parental alleles were identified by aligning them using Sequencher 3.11 software (version 3.1.1; Gene Codes, Ann Arbor, MI). Because cloning can introduce artifact sequence differences, polymorphisms were confirmed by direct sequencing of genomic DNA amplification products using a nested primer (nested primer sequences are not shown).

### Isolation of Dax1 and Dmrt1 genomic sequences

A PCR-based approach (Brunelli et al. In press) was used to isolate lambda clones of *Dax1 *and *Dmrt1 *from a λ Zap II (Stratagene, La Jolla, CA) genomic library created from an O × H F1 hybrid. Following sequencing of the *Dax1 *and *Dmrt1 *lambda library clones by PCR walking, the structure of the *Dax1 *gene was deduced based on sequence alignment with the relevant sequences of *Dicentrarchus labrax *and *Oreochromis niloticus*. Intron and promoter sequences of *Dmrt1 *were identified based on alignment of the recovered genomic clone with rainbow trout *Dmrt1 *published cDNA sequence.

### Genotyping of doubled haploid populations

#### PCR-RFLP

Non-coding sequences of the candidate genes *Sox6, Dmrt1 and Amh *have sequence substitutions between alleles of the different parental lines that alter recognition sequences of restriction enzymes (RE). Utilizing these differences, doubled haploid fish of the mapping family were genotyped for the parental allele containing the polymorphic restriction site by PCR amplification, followed by RE digestion. Products of the RE-digest were size-fractionated in 1.5% agarose gels. Appropriate restriction enzyme digestions were done in 20 μl volume reactions according to the manufacturer's directions (New England Bio-labs, Ipswich, MA). Gene-specific primers utilized in RFLP-genotyping are listed in Table 5. Polymorphic RE-sites are listed in Table 1.

#### Taqman assay

A single nucleotide polymorphism (SNP) was detected between OSU and HC in the deduced promoter region of *Dax1*. The polymorphism did not change any published recognition sequence of a RE. A Taqman assay (Applied Biosystems, Foster city, CA) was used to score doubled haploids of the O × H mapping family for having either OSU or HC alleles. Primers and probe sequences used in the Taqman assay are listed in Table 1.

#### Microsatellites

A microsatellite repeat nested in the *Sox6 *fourth intron was found to have a different size among the different *Sox6 *alleles (Table 4). Size differences were large enough to be analyzed in a 2.5% agarose gel stained by ethidium bromide and viewed under UV light. Primer sequences used for amplifying the microsatellite are listed in Table 5. Default PCR reaction conditions were used for the amplification.

### Linkage mapping

Prior to linkage analysis, segregation data of all loci was checked for deviations from expected Mendelian ratios using a chi-square test (α = 0.05). Genotyping data of candidate genes was incorporated into the pre-constructed O × H and O × A linkage maps using Map Maker 3.0/EXP [34]. Marker orders were retained from the previous publications using these families [22,32]. For *Sox6*, doubled haploids were genotyped for receiving OSU *Sox6i *or *Sox6ii *alleles, while the Arlee *Sox6i *allele was genotyped as a null allele. The locations of newly-genotyped loci were determined using the "try" command, with the Kosambi map function, a minimum LOD score of 3.0 and a maximum theta of 0.45.

### Promoter sub-sequence analysis

A 2.2 Kb region upstream of *Dax1 *and 0.6 kb region upstream of *Dmrt1 *coding regions were sequenced from the isolated lambda clones. The promoter sequences were analyzed for the presence of putative regulatory elements for transcription factors known to be involved in sex differentiation using MatInspector software (Genomatix Software GmbH, Munich, Germany).

## Authors' contributions

MA drafted the manuscript, assisted in designing the study, recovered genomic sequences and genotyped doubled haploid fish of reference families. JB assisted in genotyping analyses and provided technical assistance. RD conducted linkage analysis on the reference families. GT conceived the study, participated in its design and coordinated the roles of the authors. All authors read and approved the final manuscript.
